# Efficacy of Ambroxol lozenges for pharyngitis: a meta-analysis

**DOI:** 10.1186/1471-2296-15-45

**Published:** 2014-03-13

**Authors:** Jean-François Chenot, Peter Weber, Tim Friede

**Affiliations:** 1 Department of General Practice, University Medicine Greifswald, Ellernholzstr. 1-2, 17487 Greifswald, Germany; 2 Hausarztpraxis am Dingbängerweg 37, 48163 Münster, Germany; 3 Department of Medical Statistics, University Medical Center Göttingen, 37073 Göttingen, Germany

**Keywords:** Pharyngitis, Sore throat, Mint flavoured lozenges, Ambroxol, Primary care, Pain relief, Meta-analysis

## Abstract

**Background:**

Ambroxol has a local anaesthetic action and is marketed for pain relief for sore throat. The objective is to examine the efficacy and safety of ambroxol for the relief of pain associated with acute uncomplicated sore throat.

**Methods:**

A systematic review of the literature and meta-analysis. Selection criteria consisted of randomized controlled trials which compared ambroxol to placebo or any other treatment for sore throat. Two reviewers independently assessed for relevance, inclusion, and risk of bias. Weighted mean differences (WMDs) were calculated and are reported with corresponding 95% confidence intervals (CIs).

**Results and conclusion:**

From 14 potentially relevant citations, five trials reported in three publications met the inclusion criteria, three of them were published twice. Ambroxol lozenges were compared in different dosages (5–30 mg) with mint flavoured lozenges and once with benzocaine. Main outcome was a ratio of pain reduction measured repeatedly over 3 h compared to baseline on 6-item verbal rating scale. A total of 1.772 adult patients participated in the trials. Pain intensity decreased in both study arms. A meta-analysis of the 5 controlled trials resulted in a difference in pain reduction compared to placebo of -0.11 (95% CI [-0.15, -0.07]; p < 0.0001) favouring ambroxol 20 mg. Quality of reporting of the studies was low. Ambroxol is slightly more effective in relieving pain in acute sore throat than mint flavoured lozenges over a period of 3 h. However, the additional benefits of ambroxol beyond three hours, remain unclear given that more than 50% of patients using mint flavoured lozenges for pain relief reported good or very good efficacy after 1 day compared to 69% with ambroxol. Ambroxol is a safe option for individual patients with mainly local symptoms asking for treatment.

## Background

Pharyngitis or sore throat is a highly prevalent mostly self-limiting condition for which most people do not seek medical attention [1]. Viral or bacterial infections causing sore throat generate pain through inflammation of the pharynx and the surrounding lymphatic tissue. Most patients with sore throat seen in primary care have viral infection and no indication for antibiotics. Antibiotic treatment may shorten the duration of symptoms in a bacterial throat infection (from 3.3 to 2.7 days), the benefits are considered moderate [2]. Physicians frequently assume that patients seeking care expect a course of antibiotics. It has been shown, that pain relief is more important for patients and patients who desire antibiotics may in fact want treatment for pain [3].

Gargling, drinking warm liquids and oral antipyretic or analgesic drugs are common supportive treatments [4]. Ambroxol lozenges are marketed in many countries worldwide for pain relief for sore throat. The local anaesthetic action of ambroxol, a sodium channel blocker, might be effective to relieve symptoms due to inflammation [5-7]. Therefore ambroxol might represent a useful option to meet patients’ needs and avoid unnecessary prescription of antibiotics [8].

We performed a systematic review and meta-analysis of the effects of ambroxol to relief pain of sore throat compared to placebo in outpatients and discuss the implications for practice.

## Methods

This is a meta-analysis conducted according to the guidance of the PRISMA statement [9].

### Data sources

We searched in the three following electronic bibliographic databases: MEDLINE, EMBASE and Central of Cochrane Data Base of systematic reviews. We included studies published between 1966 and May 31, 2011. The search algorithm contained the following keywords and MeSH-terms: [Ambroxol AND (pharyngitis OR tonsillitis OR rhinopharyngitis OR tonsillopharyngitis OR pharyngotonsillitis OR sore throat OR pharynx* OR tonsil*)]. Additionally, we searched manually through the reference list of the identified articles. We also contacted the manufacturer of Ambroxol and searched ClinicalTrials.gov [10] for registered, but otherwise unpublished trials.

### Study selection

Eligibility criteria: Our search included published randomized controlled trials (RCTs) that compared ambroxol as treatment for sore throat with a placebo. We did not place restrictions on eligibility according to drug dosing, duration of application or publication language and did not exclude specific populations or age groups.

Screening process: Two independent reviewers (JFC, PW) screened the citation titles and abstracts using a predesigned orm. We excluded titles and abstracts that clearly did not meet the inclusion criteria. For those titles fulfilling inclusion criteria full-text articles were obtained. The two reviewers resolved disagreements by consensus.

### Data extraction and analysis

We extracted information from the original reports onto standardized forms. All data was entered into Review Manager (RevMan) [11]. The primary outcome was a time and baseline adjusted value for reduction of pain intensity on a verbal rating scale ranging from 0 to 5. Measurements were done at baseline after 30, 60, 120 and 180 minutes. The values were subtracted from the baseline (Pain intensity difference PID) and area under curve (AUC) and adjusted for time with the following formula: AUC = 0.5 × PID30 + 0.5 × PID60 + PID120 + PID180. This AUC divided by three was reported as the primary outcome [12]. A value of -1.0 represents full pain reduction over 3 hours and a value of – 0.1 represents correspondingly 10% pain reduction over 3 hours. The secondary outcome was patients‘ evaluation of overall efficacy with a 4-point verbal rating score (“very good”, “good”, “not so good”, “poor”) at the end of each treatment day. We also extracted data on reported adverse events. We did not have access to individual data and used summary data provided in the publications.

We used the Cochrane Collaboration tool for assessment of the risk of bias [11]. The assessment was done independently by (JFC, TW). There were no disagreements. We used a fixed-effect model to combine the treatment effect estimates from the individual studies with inverse variance weighting. The combined estimate of the treatment effect is reported with 95% confidence interval. To facilitate interpretation of the clinical relevance of the treatment effect on the primary outcome we expressed the treatment effect also in terms of the probability that a patient treated with ambroxol achieves a greater or faster pain reduction within three hours than when treated with the control. This effect measure is known as the probabilistic index or relative effect and was suggested for the assessment of clinical relevance [13]. For the conversion between effect measures we assumed that the distribution of the primary outcome is approximately normal, which seems justified since the outcome measure is a summary statistic of several measurements. However, the individual pain intensities are not required to follow a normal distribution. Between-study heterogeneity was assessed using I^2^ measure and a formal hypothesis test. Forest plots showing the effect estimates of the individual studies and the combined effect allow visual assessments of the heterogeneity and provide an overview of the results. Subgroup analyses were carried out for different doses of ambroxol. We tested for subgroup differences and report the p-values.

## Results

### Search results and study selection

**Figure 1:**
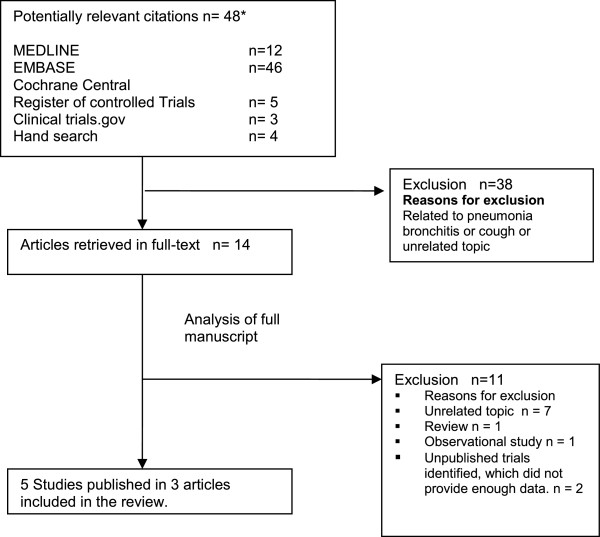
**Literature search results.** * due to duplicate identification in different databases numbers do not add up to 48.

Overall 14 potentially relevant publications were initially identified. We extracted five relevant RCTs reported in three publications [14-16]. Three RCTs were published twice [14,16]. One publication summarized all previously published trials providing additionally data from two so far unpublished trials in a so called EBM-based clinical documentation [15]. We identified additionally two unpublished registered and completed RCTs in the study registry ClinicalTrial.gov .These did not provide enough data to be included in the meta-analysis. All trials were financed by the manufacturer of ambroxol lozenges. The search process is described in Figure 1.

### Study characteristics and assessment of reporting

The characteristics of the included five trials are given in Table 1. There were a total of 1,772 adult patients randomized in five trials. All trials compared 20 mg ambroxol with placebo. There was a dose finding study comparing additionally 5 and 10 mg ambroxol [15] with placebo and two studies had a treatment arm with 30 mg ambroxol [14,16]. One study included a treatment arm with the local anaesthetic benzocaine, but outcomes were not reported [15].

**Table 1:** Characteristics of the included randomized controlled trials and two incompletely reported trials

**References**	**Number of participants**	**Age (yr)**	**Setting (country)**	**Treatment**	**Comparison**	**Follow up**	**Dropouts**
de Mey et al. 2008 [15]	92	n.r.*	outpatients (n.r)	Ambroxol 5, 10, 20 mg	Placebo	1 day	11
Schutz et al. 2002^,^[14]	215	39,4 ± 15	outpatients (Germany)	Ambroxol 20 mg	Placebo	2 days	19
Fischer et al. 2002 [16]	331	37 ± 13	outpatients (RSA)	Ambroxol 20, 30 mg	Placebo	3 days	48
Fischer et al. 2002 [16]	383	36 ± 12	outpatients (RSA)	Ambroxol 20, 30 mg	Placebo	3 days	58
de Mey et al. 2008 [15]	751	n.r.*	outpatients (n.r.)	Ambroxol 20, 3 mg benzocaine**	Placebo	3 days	27
**Registered trials with incompletely reported results**							
clinicaltrials.gov 2006	221	n.r.	outpatients (RSA)	Ambroxol 20 mg	Placebo	3 days	0
clinicaltrials.gov 2008	259	n.r.	outpatients (China)	Ambroxol 20 mg	Placebo	n.r.	0

n.r. = not reported, RSA = Republic of South Africa, * inclusion age was 16 to 80 years, but average age for those trials was not reported **the outcome of the benzocaine treatment arm is not reported.

Inclusion criteria were sore throat not lasting longer than three days before inclusion in the study. Patients with suspected bacterial infection were excluded on the basis of clinical findings absence of seropurulent or fibrinous exudates. There are inconsistencies in the reporting participants’ age as inclusion criterion. Throat swabs were not taken. Although it is specified that all patient were outpatients it remains unclear if patients were recruited in ambulatory care, emergency departments or especially set up clinics.

Risk of bias is presented in Table 2. The publications do not meet the CONSORT statement standards of reporting [17]. For instance, for only two trials patient flow charts were reported [16]. From these two charts it appears that all screened patients were randomized, which is rather unusual for a clinical trial setting and is therefore casting doubt on the accuracy of reporting. The number of eligible patients screened is not shown for any of the other trials included in this review. Age of the included patients cannot be assessed for the two newly reported trials in the summary report [15]. Data was analysed as intention to treat (ITT) and per protocol (PP) with last observation carried forward (LOCF). There is no evidence of selective drop out. No power or sample size calculations were reported.

**Table 2:** **Bias assessment of included trials**[9]

**Reference**	**Adequate sequence generation**	**Allocation concealment**	**Blinding of participants and personnel**	**Blinding of outcome assessment**	**Incomplete outcome data adressed**	**Free of selective reporting**	**Free of other bias**
de Mey et al. [15]	Yes	Unclear	Yes	Unclear	Yes	Unclear	No
Schutz et al. [14]	Yes	Unclear	Yes	Unclear	Yes	Unclear	No
Fischer et al. [16]	Yes	Unclear	Yes	Unclear	Yes	Unclear	No
Fischer et al. [16]	Yes	Unclear	Yes	Unclear	Yes	Unclear	No
de Mey et al. [15]	Yes	Unclear	Yes	Unclear	Yes	No	No

### Primary outcome

Reported pain reduction from baseline over a period 180 minutes ranged from:

• 37 to 42% from ambroxol 20 mg

• 40 to 49% from ambroxol 30 mg

• 27 to 35% from mint flavoured placebo lozenges.

**Figure 2:**
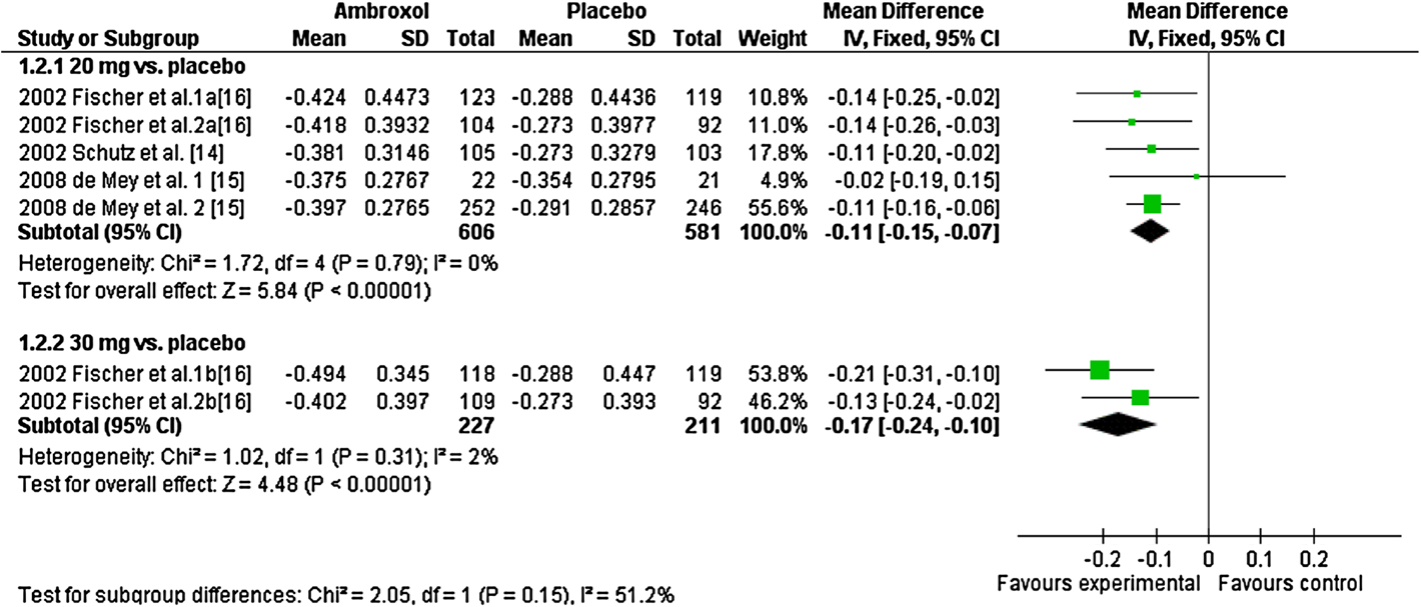
Forest plot of primary endpoint comparing Ambroxol 20 mg and 30 mg versus placebo.

The Forest plots show the difference of pain reduction of ambroxol 20 mg and 30 mg over 3 h compared to placebo (Figure 2). The summary of the observed difference of pain reduction is -0.11 (CI_95_ [-0.15; -0.07]) for 20 mg and -0.17 (CI_95_ [-0.24; -0.10]) for 30 mg of ambroxol. Although it seems that Ambroxol 30 mg is more effective than 20 mg the difference is statistically not significant (p=0.15). The standardized mean differences are -0.34 (CI_95_ [-0.46; -0.23]) and -0.43 (CI_95_ [-0.62; -0.24]) for 20 mg and 30 mg of ambroxol, respectively. These translate into probabilities of 59% and 62% that the pain reduction is greater or faster with ambroxol compared to placebo. The two unpublished trials also reported statistically significant differences in pain relief favouring ambroxol 20 mg, but did not provide enough data to be included in this meta-analysis.

### Secondary outcome

As secondary outcome patients’ evaluation of overall efficacy regarding efficacy was measured in all studies. Data of three trials were individually published [14,16]. Data of two studies were not published individually [15]. The results of all five trials were pooled in the publication summarizing all five trials [15]. In total 69% in the 20 mg Ambroxol group compared to 53% in the Placebo group rated the efficacy of the treatment as good or very good at the end of the first day. At the end of day 2 and 3 results were 78% to 59% and 83% to 67% respectively. In two studies treatment only lasted 1 and 2 days respectively [14,15]. The available data did not allow a calculation of confidence interval for the reported differences.

### Adverse events

Adverse events and number of patients with at least one adverse event were reported in all trials. Two trials reported adverse advents only for the 20 mg form and for the placebo group but not for other dosages or benzocaine [15]. There was inconsistency in the report of the number of adverse events in the summarizing publication. In total 20.5% of the patients treated with 20 mg Ambroxol had adverse events compared to 11.9% in the Placebo group. Adverse events were mild such as oral hypoaesthesia, dysgeusia or pharyngeal hypoaesthesia [15], dry mouth, skin rash, nausea, insomnia [16], increased sweating, pharynx oedema, migraine, tremor and pharyngitis [14]. Most of them were more likely due to symptoms related to progression or complication of underlying disease such as upper respiratory infection. In one trial the number of patients who discontinued treatment is unclear [15].

## Discussion

The results of this systematic review show that ambroxol lozenges are consistently more effective for local pain reduction in adult patients with sore throat compared to a mint flavoured placebo within 3 h. Overall treatment effects were more often rated as good or very good on a 4-point Likert scale compared to placebo after one, two or three days. More than 50% reported of patients in both groups reported effective pain reduction (“very good” or “good”) after 1 day. The observed adverse effects although more frequent in the treatment arms were usually mild or could be attributed to the medical condition. All patients were selected based on clinical presentation to minimize the presence of streptococcal throat infection and did not receive concomitant antibiotics. This is in line with recommendations from most European guidelines [18,19].

Sucking candy is a popular home remedy and it is likely that sucking lozenges is not just a placebo but decreasing pain e.g. by increasing saliva flow and reducing dryness of the oral mucosa. Additionally menthol is a pharmacologically effective ingredient known to affect sensation of the oral mucosa [20].

The primary outcome as defined in the studies cannot be interpreted easily in clinical terms. Therefore, it is unclear what should be considered a minimal important difference (MID) on that scale for sore throat. The summarized observed pain reduction of -0.11 (CI_95_ [-0.15; -0.07]) for 20 mg ambroxol suggesting roughly 10% more pain reduction compared to placebo after 3 hours seems small. Ambroxol is available as 20 mg preparation.

Sore throat is usually a self-limiting condition lasting on average for 6 to 8 days with decreasing intensity [21]. For clinically relevant treatment effects beyond 3 h we have to rely on the secondary outcome the patients’ global assessment of efficacy after one and three days. However, these data were only presented by treatment arm pooled across trials [22] which prevents the application of appropriate meta-analytic methods which requires an analysis stratified by study. The reported differences for patient evaluation of overall efficacy after 1, 2 and 3 days varying from 13 to 16 percentage points [22] need therefore be interpreted cautiously because of methodological shortcomings. Also the effect appears to be rather small with more than 50% reported very good or good efficacy with both treatments after 1 day.

The effect of local treatments naturally wears off after a few hours. Summarized data on repeated use of ambroxol or mint-flavoured lozenges is provided but does not allow drawing conclusion on effectiveness related to continuous use of lozenges. There are many alternative local and systemic treatments for sore throat available [4,23]. Due to different patient populations and different outcome-measures efficacy cannot be compared directly. In patients with associated systemic symptoms like arthralgia, headache and chills, over the counter analgesic medications with systemic action like Paracetamol (Acetaminophen) or Ibuprofen might to be a better treatment option. Additional benefits of ambroxol lozenges for local pain relieve for those patients has not established. Concomitant use of such medication was prohibited in the reported trials, but it is reported that some patients used additionally other non- specified remedies.

There are many alternative local and systemic treatments for sore throat available [4,22]. Due to different patient populations and different outcome-measures efficacy cannot be compared directly. In patients with associated systemic symptoms like arthralgia, headache and chills, over the counter analgesic medications with systemic action like Paracetamol (Acetaminophen) or Ibuprofen might to be a better treatment option. Additional benefits of ambroxol lozenges for local pain relieve for those patients has not established. Concomitant use of such medication was prohibited in the reported trials, but it is reported that some patients used additionally other non- specified remedies.

Case reports of adverse effects related to systemic ingestion of Ambroxol have been published [24-26], however in this large sample no serious side effects from mainly topical application of ambroxol were observed.

### Limitations

There are some limitations to this systematic review. We had to rely on published data and had no access to individual patient data. We cannot exclude publication bias in favour of trials finding ambroxol to be beneficial. It is not likely that the two trials which could not be included in the review would have changed the estimate of efficacy significantly since they also found ambroxol more effective than mint-flavoured lozenges. Other limitations are related to the trials themselves and how they were reported.

The RCTs were all sponsored by the manufacturer and did not meet current standards of reporting. A patient flow chart as stipulated by the CONSORT-statement [17] was only available for two trials [16]. Some data such as the country where the study was conducted was not reported for all trials. The settings where patients were recruited are not sufficiently described. Selection bias is very likely since none of the included trials report the number of screened patients for eligibility. There were only few dropouts and it is not reported whether patients received some kind of incentive for participation and completion of the study.

## Conclusion

Ambroxol is slightly more effective in relieving pain in acute sore throat than mint flavoured lozenges over a period of 3 h. However, the additional benefits of ambroxol beyond three hours remain unclear given that more than 50% of patients using mint flavoured lozenges for pain relief reported good or very good efficacy after 1 day. Ambroxol is a safe option for individual patients with mainly local symptoms asking for treatment. In patients with associated systemic symptoms over the counter analgesic medications might be a better option.

## Competing interests

The authors (PW, TF) declare that they have no proprietary, financial, professional or other personal interest of any nature or kind in any product, service and/or company that could be construed as influencing the position presented in this paper. Jean-Francois Chenot hat acted in 2009 and 2010 as consultant for Böhringer Ingelheim the manufacturer of ambroxol lozenges.

## Authors’ contribution

The literature search and data extraction was done by JF and PW. Statistical analysis was done by JFC and TF. All authors drafted and reviewed the manuscript. JFC is a General Practitioner and health scientist, PW is a resident in Internal Medicine and TF is a biostatistician. All authors read and approved the final manuscript.

## Pre-publication history

The pre-publication history for this paper can be accessed here:

http://www.biomedcentral.com/1471-2296/15/45/prepub
